# Accuracy in determining interproton distances using Nuclear Overhauser Effect data from a flexible molecule

**DOI:** 10.3762/bjoc.7.20

**Published:** 2011-02-01

**Authors:** Catharine R Jones, Craig P Butts, Jeremy N Harvey

**Affiliations:** 1 Department of Chemistry, University of Bristol, Cantock’s Close, Bristol, BS8 1TS, United Kingdom

**Keywords:** conformation, internuclear distances, NMR spectroscopy, NOE

## Abstract

The determination of accurate NOE-derived interproton distances and confirmation/prediction of relative populations in multi-conformer, flexible small molecules was investigated with the model compound 4-propylaniline. The low accuracy assumed for semi-quantitative NOE distance restraints is typically taken to suggest that large numbers of constraints need to be used in the dynamical analysis of flexible molecules, and this requires, for example, the measurement and Karplus-type analysis of scalar coupling constants (^3^*J*_CH_ and ^3^*J*_HH_). Herein we demonstrate that, contrary to this common perception, NOE measurements alone are accurate enough to establish interproton distances, and hence conformational detail, in flexible molecules to within a few percent of their ensemble-averaged values, hence reducing the demand for additional restraints in such dynamic analyses.

## Introduction

Information obtained from Nuclear Overhauser Effect (NOE) experiments in NMR spectroscopy is widely employed in the determination of stereochemical and conformational details [1]. It is traditionally used in a qualitative or semi-quantitative manner to establish gross differences between conformers. However, the quantitative use of NOEs is often discounted or at least considered to be only approximately accurate. This perceived inaccuracy in NOE-distance relationships arises because many factors may perturb NOE intensities, including spin diffusion, selective polarisation transfer, variation in *τ*_c_ between spins, accuracy of signal integration and conformational flexibility [1]. Despite this, we have recently shown that surprisingly accurate NOE-derived distances can be obtained in small organic molecules, and that many of the perturbing factors do not contribute significantly to NOE intensities when the molecule of interest is in the fast tumbling regime and measurements are made within the Initial Rate Approximation limits [2]. In this previous report we observed mean errors of ~3% in distances obtained from 1D NOESY experiments on the rigid molecule strychnine in *d*_6_-benzene (as compared to their computationally-derived values [3]). With accurate distances obtained in a rigid organic molecule, it seems sensible to examine whether this approach can be extended more generally to multi-conformational systems with similar accuracy. Further, it is likely that the accurate interproton distance-assessments from NOE will allow accurate modelling of conformer populations in solution with fewer NOE constraints, or indeed improvements in the accuracy of modelling with the same number of NOE constraints.

If flexible systems exhibiting multiple conformations in solution are interconverting rapidly on the NMR time-scale, then conformational exchange will lead to ensemble-averaging of the observed NOEs for each corresponding interproton distance in each contributing conformer. One approach to analysing such ensemble-averaged NOEs is to assume the molecule will occupy a number of distinct low-energy conformations with particular populations in solution. The ensemble-averaged NOE-determined distances can thus be used, along with computations of conformer geometries, to confirm the structures and energies/populations of contributing conformers. An excellent description of the advantages and disadvantages of such an approach was made by Kozerski et al., using conformational ensemble fitting to NOE data from 2,3-dihydrobenzofuran derivatives to determine stereochemical and conformational information [4]. Critically, they highlight the challenge in fitting multi-conformer, multi-isomeric models, which require large numbers of NOE contacts in order to extract the best-fit to these loose data. On the other hand, with more accurate distances available, we suggest it will be possible to identify not only geometry, but also fit conformation populations to within reasonable errors.

## Method

The determination of interproton distances from NOE data previously described by us is based on comparison of relative NOE intensities for pairs of spins in 1D transient NOESY experiments [2]. Assuming that the molecule of interest is in the fast tumbling regime and that the Initial Rate Approximation holds true, the normalised NOE intensity between two spins *I* and *S*, η*_IS_*, is proportional to the cross-relaxation rate, σ*_IS_*, between these spins and the mixing time, *τ*_m_, of the experiment (Equation 1). In turn, the cross-relaxation rate, σ*_IS_*, between spins *I* and *S* is proportional to the internuclear distance between spins I and S (r*_IS_*^−6^) as described in Equation 2. A more complete description of these equations and their use in determining interproton distances can be found in references [1] and [2].

[1]
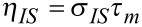


[2]
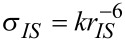


where


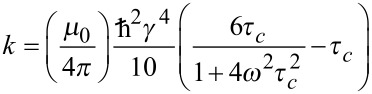


Assuming that the values defining *k* (ω-Larmor frequency, *τ*_c_-rotational correlation time, γ-magnetogyric ratio) remain constant for *each spin pair in a given selective inversion experiment*, the ratio of intensities of a pair of NOE signals, η*_I_*_1_*_S_*:η*_I_*_2_*_S_*, *within that experiment* can thus be assumed to be proportional to the ratio of their internuclear distances (Equation 3). Thus, by comparing η*_I_*_1_*_S_* and η*_I_*_2_*_S_* *within the same selective inversion experiment*, we only need to know one distance, e.g., r*_I_*_1_*_S_*, in order to calculate the second distance, r*_I_*_2_*_S_*.

[3]
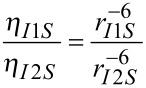


As outlined above, when there are multiple conformations describing a flexible molecular system, the matter of internuclear distance determination becomes more challenging. A general outline of the treatment of flexible small molecules using NOE experiments for both conformational and population analysis can be found in reference [1]. Larger flexible systems with multiple conformations such as peptides and proteins have been investigated using NOE-derived data, where techniques such as ensemble-averaged full relaxation matrix approaches are used to simulate NOEs [5–7]. The relative proportions of the various conformations contributing to the ensemble are then iteratively adjusted to derive the best fit to the experimentally measured NOEs. Accurate determination of populations in these analyses has always been limited by the inherently low accuracy of the NOE-derived restraints used, resulting in a broad range of conformer populations fitting the observed NOE restraints.

On the other hand, with the high accuracy provided by the NOE-distance analysis we employ, we have recently identified and quantified a previously unrecognised conformer of strychnine through a relatively minor deviation in a single measured NOE-derived interproton distance across the seven-membered ring of strychnine [8]. This intra-ring distance was observed to be ~0.6 Å (15%) shorter than expected by X-ray crystallography [9] and DFT [3], which would traditionally be considered an acceptable ‘experimental error’ for NOE-derived interproton distances. However, given that the average errors for NOE-derived distances in our earlier study [2] were ~3%, this was identified as significantly anomalous. In the event, a second low-energy conformer was identified with a population of ~2.2% compared to the major conformer. By incorporating this second conformer, the error for the problematic distance was reduced from 15% to 3%, while no other significant changes in the NOE-derived distances arose.

We now sought to investigate the conformer populations of a flexible molecule where conformational exchange causes the perturbation of more than one distance, and hence more than one NOE intensity. Herein, we report that the high accuracy reported for relatively rigid molecules is maintained for multiple NOE-derived distances arising from conformationally flexible molecules when compared to their time-averaged computationally-derived distances in the alkyl chain of a small molecule – 4-propylaniline. These NOE-derived distances can thus be applied to modelling the populations (and hence energy differences) of the conformers, again with good accuracy when compared to their calculated values.

## Results and Discussion

**Figure 1:**
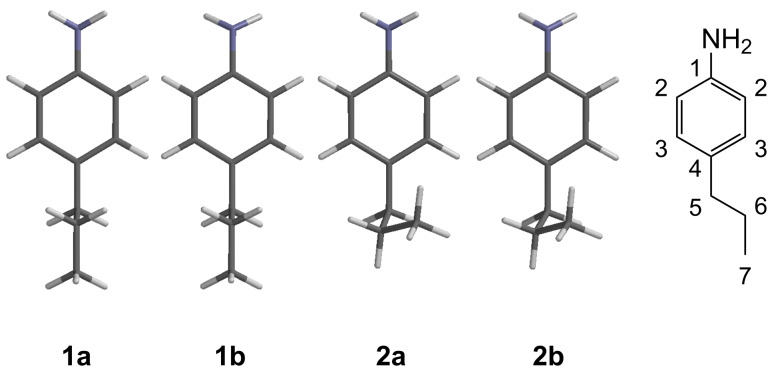
Four low energy conformations of 4-propylaniline obtained from B3LYP/6-31G* conformational search and labelled 2D structure (**1a**/**2a** differ from **1b**/**2b** in the pyramidalization at the amine nitrogen, see main text for details).

A B3LYP/6-31G* conformational search of 4-propylaniline leads not surprisingly to four non-degenerate low-energy conformers, with the propyl chain either in an anti, **1a** and **1b**, or a gauche, **2a** and **2b**, conformation (Figure 1). The anti conformers, **1a** and **1b**, differ from each other only in the opposite pyramidalisation of the amino group: The gauche conformers **2a** and **2b** are similarly related, with two further degenerate gauche conformers of **2a** and **2b** arising by rotation of 120° around the C5–C6 bond. In each case, the isomer ‘**a**’ is the one in which the aniline H atoms are on the same side of the benzene ring as the carbon chain. The optimised H–H distances in **1a** are very similar to those found in **1b**, and likewise for **2a** and **2b**.

The calculated energies of conformers **1a** and **1b** differ by less than 0.01 kJ/mol from each other, and lie only 1.99 and 1.94 kJ/mol lower than **2a** and **2b**. After correction for zero-point energy and thermal and entropic corrections, the relative calculated ΔG at 298 K for conformers **1a**, **1b**, **2a** and **2b** were 0.09, 0.00, 3.20 and 3.14 kJ/mol, respectively. Finally, on including the solvation free energies (in CDCl_3_) for all species, this leads to corresponding predicted free energies of 0.10, 0.00, 3.55 and 3.39 kJ/mol, respectively. As the B3LYP relative electronic energies are not expected to be highly accurate, single-point calculations using two very accurate local correlated methods were used to refine the gas phase relative energies. At the LCCSD(T0) level of theory, relative electronic energies of 0.07, 0.03, 0.09 and 0.00 kJ/mol, respectively, are obtained. The better description of dispersion interactions stabilises the more compact gauche conformers as expected. LPNO-CEPA-1/cc-pVTZ calculations, yield relative energies of 0.03, 0.00, 0.20 and 0.14 kJ/mol, very close to the coupled-cluster values, suggesting that these quantities are reliable to ± 0.5 kJ/mol and perhaps better.

Including the corrections for zero-point energy, thermal and entropic corrections, and solvation, these correlated ab initio calculations lead to predicted relative free energies in solution for the four conformers of 0.15, 0.00, 1.63 and 1.43 kJ/mol based on the LCCSD(T0) calculations, and very similar values of 0.14, 0.00, 1.76 and 1.60 kJ/mol based on the CEPA calculations. Taking into account the two-fold degeneracy of each of the gauche conformers **2a** and **2b**, this gives expected equilibrium populations of 23, 24, 25 and 27% or 24, 25, 25 and 26%, respectively. As the distances in each pair of conformers are so similar, this can be described more concisely as being predicted populations of **1 (a + b)** and **2 (a + b)** of 47% and 53% or 49% and 51%.

Experimentally, a selection of interproton distances: H3–H5, H3–H6, H3–H7, H5–H7, was determined using NOE intensities from 1D NOESY experiments. The intensities of the measured NOE signals were first corrected for the chemical equivalence/symmetry in each group by dividing the NOE intensity between signals *I* and *S*, η*_IS_*, by n*_I_*n*_S_*, where n*_I_* and n*_S_* are the number of chemically equivalent spins in the groups giving rise to signals I and S respectively [1]. In order to convert the corrected NOE intensities into physically realistic interproton distances, vide supra, they need to be scaled against a single fixed reference distance for which an NOE has also been measured [1–2]. The obvious candidate distance, between the fixed aromatic H2 and H3 protons, could not be used as the experimental NOE signal between these two protons showed strong coupling artefacts that obscured the NOE intensity. Instead, the NOE data were internally calibrated from the conformationally averaged H3–H5 NOE, assuming a reference distance for this proton pair of 2.77 Å (which is the calculated ensemble-average using the populations for **1a/b** and **2a/b** as determined above). The relative NOE intensities were then converted to ensemble-averaged internuclear distances, r_NOE_, by applying the r^−6^ analysis vide supra. Where the same ensemble-averaged interproton distance was measured by two NOE experiments, e.g., *H3*–H6, *H6*–H3 (where the labels in italics are the inverted proton respectively) the experimental distance, H3–H6, was taken as the average of the pair and the results are presented in Table 1.

**Table 1:** NOE-derived and ensemble-averaged interproton distances.

	r_NOE_ (Å)	r_calc_ (Å)	% error

H3–H5 (ref)	2.77	2.77	-
H3–H6	3.21	3.34	3.83
H3–H7	4.16	4.05	2.54
H5–H7	3.13	3.08	1.67

To calculate the effective interproton distances, r_calc_, for the conformationally averaged molecule, i.e., those distances which theoretically should be determined by the NOE experiments, each interproton distance, r*_IS_*, as determined in the B3LYP/6-31G* structural optimisation for the conformers **1a**, **1b**, **2a** and **2b** was converted into a corresponding NOE intensity for each conformer using the r^−6^ relationship (η*_IS_* = r*_IS_*^−6^). The measured NOEs all involve protons in chemically equivalent groups, e.g., H3 and H3’ or H6 and H6’, so the effective distances were obtained from the calculated structures (and thus relative NOE values determined) for *all* the contributing chemically equivalent pairs in each case, e.g., the H3–H6 NOE derived from r_H3–H6_, r_H3–H6’_, r_H3’–H6_ and r_H3’–H6’_, which were combined with <r*_IS_*^−6^> = (Σr*_IS_*^−6^)/(n*_I_*n*_S_*) ^,^ where n*_I_* and n*_S_* are the number of equivalent spins in the groups *I* and *S*, respectively. These individual NOE intensities for each of the six conformers were then weighted using the calculated Boltzmann populations from above, to convert them into effective distances.

A comparison of the resulting NOE-derived distances, r_NOE,_ and ensemble-averaged distances, r_calc_, from calculations for H3–H6, H3–H7 and H5–H7 are shown in Table 1. The observed errors for these distances derived from a free-rotating group are in the range of 1–4% (mean 2.68%) and this compares very well with the observations in our previous work on the rigid strychnine system, which gave mean errors of ~3% in *d*_6_-benzene and ~4% in CDCl_3_ [2–3].

**Figure 2:**
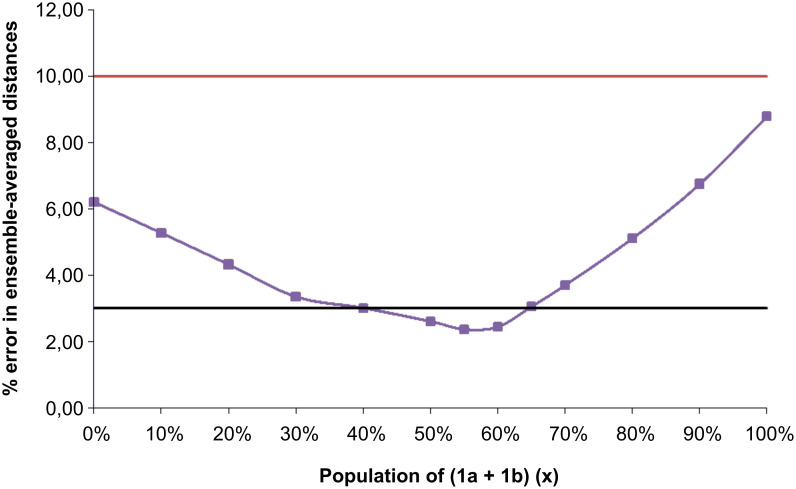
Plot of the mean calculated error arising from fitting the population distribution of conformers **1a/b** (**x**) and **2a/b** (1−**x**) to the experimental NOE-derived interproton distances. Exemplar error thresholds are represented at 3% (black line) and 10% (red line).

An alternative method of data analysis might assume that the NOE measurements and the DFT conformer structures are accurate, but that the computed free energies (and hence the populations) are not. Thus, one might use the NOE and geometry data to model the conformer populations and hence the relative energies. In theory, there are too many conformers (four non-degenerate) to model their populations against the measured NOEs. However, if one assumes the degeneracy of **1a/b** and also **2a/b**, on near-symmetry grounds, then the relative populations of conformers **1a** and **1b** would be equal, (**x**), as would **2a** and **2b**, (1−**x**), reducing the problem to a single unknown with three NOE restraints. Figure 2 shows the plot of the average errors, compared to experiment, arising from weighting the calculated NOE intensities (vide supra) for each conformer by a range of populations (**x**) of **1a/b**. The best fit to experiment is obtained at ~55% **1a/b**, (and thus ~45% **2a/b**), corresponding to a free energy difference of ca. 2.1–2.2 kJ/mol between conformers **1a/b** and **2a/b**. This compares extremely well with the highest level calculations, which suggest a corresponding free energy difference of ca. 1.5 kJ/mol. This remarkable match in populations and energies supports the proposition that fitting populations of conformers from the NOE-derived distances is an inherently accurate approach when the geometries of the contributing conformers can be accurately described.

Figure 2 also demonstrates the requirement for the relatively accurate determination of internuclear distances in order to fit conformationally flexible systems. Assuming a mean 3% error in the interproton distances determined by NOEs (black line in Figure 2), the experimental results are consistent with a population of **1a/b** lying in the range 40 to 65%. However, typical semi-quantitative NOE studies are assumed to lead to much larger errors in distances, in the order of ± 10% (red line in Figure 2), or even assign distance-ranges to NOE intensities such as strong (2–3 Å), medium (3–4 Å) and weak (> 4 Å). The entire population range of conformers **1a/b** falls below the red line in Figure 2, showing that such loose assumptions of NOE accuracy would effectively allow *any* population of **1a/b** and **2a/b** to be fitted acceptably to the experimental data, requiring other constraints to be invoked in order to model even this simple system.

In summary, these results suggest that the methods we have previously described for extracting accurate interproton distances from NOE data in rigid systems can be extended with little, or no, loss of accuracy to relatively flexible small molecules. The high accuracy of this distance data allows it to be applied to assessing/confirming the relative populations of contributing conformers in small, flexible molecules with reasonable certainty, even where very few restraints are available – succeeding where traditional semi-quantitative NOE analysis would not.

## Experimental

NMR samples were prepared in 5 mm tubes with 0.7 ml CDCl_3_ and ~10 mg 4-propylaniline, in air without degassing. NMR data were all collected on a 500 MHz Varian VNMRS DirectDrive spectrometer equipped with an indirect observe probe. 1D selective transient NOESY spectra (64 k data points, 8 kHz spectral width, 500 ms mixing time, 4.096 sec*.* acquisition time, 1 s relaxation delay, 512 scans (45 minutes/irradiation)) were obtained using the Varian Chempack NOESY1D sequence which is based on the DPFGSENOE (double-pulse field gradient spin-echo NOE) excitation sculpted selective sequence reported by Stott et al. [10] and incorporates a zero-quantum filter element [11]. NOE build-up curves were obtained with mixing times up to 900 ms and the critical constancy of relative NOE intensities within each irradiation was confirmed (as well as the linearity of absolute NOE intensities).

Geometry optimisation at the B3LYP/6-31G* level was carried out using the Gaussian 03 package and frequencies were computed to characterise the minima and derive statistical mechanical corrections to the electronic energies. Gas-phase single point energies were then calculated at the four minima in Gaussian, with B3LYP/6-31G* and a polarisable continuum model (IEF-PCM, parameters for chloroform solvent, ε = 4.9). The LCCSD(T0)/cc-pVTZ calculations [12–13] were performed as implemented in the MOLPRO2008 package [14]. Inspection of the orbital domains for the different conformers show that a consistent set is obtained for all, hence the domain error on relative energies should be small. The LPNO CEPA-1/cc-pVTZ calculations [15] were performed using the implementation in the ORCA 2.8 package [16].
